# Perceived Barriers and Facilitators to Engagement and Behavioral Enactment in Weight Management Interventions Among Racial and Ethnic Minorities

**DOI:** 10.21203/rs.3.rs-7368225/v1

**Published:** 2025-09-16

**Authors:** Iyanuoluwa Oyedeji Oyetunji, Madison Ross Tinsley, Samuel Battalio, Angela Fidler Pfammatter

**Affiliations:** University of Tennessee at Knoxville; University of Tennessee at Knoxville; Northwestern University; University of Tennessee at Knoxville

**Keywords:** Obesity, Ethnic and Racial Minorities, Behavioral Intervention, Weight Loss, Healthy Lifestyle, Barriers and Facilitators, Remote Consultation

## Abstract

**Background:**

The prevalence of obesity, a risk factor for other chronic illnesses, in the US continues to rise and disproportionately affects racial/ethnic minorities. Several weight management interventions are now being delivered remotely to address this disparity, but little is known about whether this has increased access and efficacy of treatment among racial/ethnic minorities, especially from participants’ perspectives. We qualitatively explored perceived barriers and facilitators to engagement and behavioral enactment during weight management interventions among a nationally decentralized sample of racial/ethnic minorities in the US.

**Methods:**

Thirty-eight self-identified racial/ethnic minority participants who completed the active intervention phase of the parent EVO trial were invited to participate in interviews. Interviews were conducted via Zoom using a guide with open-ended questions. Transcripts were analyzed using NVivo, codebook was developed a priori, but was updated with inductive codes emerging from analysis. Themes were categorized using an adapted version of the Social Determinants of Health and further subgrouped as barriers or facilitators.

**Results:**

Twenty-six participants completed interviews between April and August 2024. Participants were mostly female (88.5%), non-Hispanic (76.9%), and Black/African American (53.8%), with a mean (SD) age of 50.3 (10.1) years. Participants identified cultural, environmental, social, personal, and study-related factors as important to successful engagement in the intervention. Some participants’ unique identities, such as being immigrants, impacted the ease of engaging with study components, including diet self-monitoring. Some factors perceived as barriers by some participants were perceived as facilitators by others.

**Conclusions:**

Perceived barriers and facilitators to successful engagement in weight management interventions are complex and are likely impacted by personal needs and contexts of participants. This underscores the need for multifaceted interventions.

## INTRODUCTION

The prevalence of obesity is increasing in the US, and the prevalence of severe obesity disproportionately affects racial and ethnic minority populations as compared to the non-Hispanic White population [1–3]. Obesity is a known risk factor for other severe cardiometabolic conditions such as hypertension, atherosclerosis, type 2 diabetes mellitus, and sleep disorders [4] and constitutes the most expensive healthcare spending on a single health condition [5, 6].

To date, weight management interventions have not realized success in closing the widening gap of obesity disparity. Inequitable burdens of costs and access associated with traditional in-person weight loss programs, such as the popular Diabetes Prevention Program (DPP) [7], have spurred a shift to remotely delivered interventions. Remotely delivered evidence-based interventions have the potential to reach more minoritized populations [8, 9], yet little has been done to evaluate whether such a shift truly increases access to intervention while promoting efficacy of treatment, especially for racial/ethnic minorities.

In addition to the disproportionate burdens of currently available treatments, other factors may be implicated in the persistent disparities in obesity prevalence [10, 11]. Socioeconomic status and educational attainment, for example, are strongly associated with health outcomes related to obesity [12, 13]. However, there is emerging empirical evidence that places greater significance on other social determinants of health (SDOH), particularly environmental level factors such as the social and built environment [14, 15]. Recognizing the important role of SDOH in the persistence of health inequities, the World Health Organization published an SDOH conceptual framework that can guide research on factors that contribute to health [16]. Thus, the purpose of the present study was to qualitatively explore perceived barriers and facilitators to engagement and behavioral enactment, guided by the SDOH framework, among those who identified as a racial or ethnic minority during a clinical trial of remotely delivered weight management interventions.

## METHODS

### Study Design and Settings

We conducted individual qualitative interviews remotely with self-identified racial/ethnic minority participants from the parent EVO trial [17], a study that compared two remotely delivered weight management interventions for 6 months and followed participants for a year. The EVO trial protocol is published elsewhere [17]. Interviews elicited perceived barriers and facilitators to engagement in the trial and the intervention and behavioral enactment.

### Participants and Recruitment

A total of 26 participants from the EVO study were purposively recruited for this study. Individuals were eligible if they had completed the active intervention phase of the study and if they self-identified as belonging to one or more federally recognized racial/ethnic minority groups: American Indian or Alaska Native, Asian, Black or African American, Native Hawaiian or Other Pacific Islander, or Latino or Hispanic. Participants provided signed informed consent on a REDCap form delivered via email. Each participant was compensated with an $80 gift card for completing the interview.

### Data Collection

Semi-structured in-depth interviews were conducted via Zoom (Version 6.0), with durations ranging from 60 to 90 minutes. All interviews were recorded and transcribed verbatim on Zoom and verified by a research assistant. The interview guide included open-ended questions eliciting insights into how specific social, cultural, and environmental factors shaped their treatment experiences, the most significant obstacles encountered during the treatment, and what aspects of their circumstances or characteristics made them well-suited for the treatment. The interview guide was collaboratively developed by SB, AP, and IO. The EVO study protocol, including all consent procedures, was approved by the Northwestern University Institutional Review Board and the University of Tennessee via a reliance agreement.

### Data Analysis

All interview transcripts were analyzed thematically using NVivo 14 [18]. The codebook was developed through an iterative process, beginning with a set of a priori codes based on the interview guide. Additional inductive codes were generated from emerging themes identified during an initial review of selected transcripts. The codebook was refined using a double-coding process: IO and MRT independently coded 10 transcripts, met to resolve discrepancies, and revised the codebook accordingly. The two investigators double-coded the remaining transcripts to ensure reliability. MRT then queried the dataset to generate code reports, reviewed the reports, and composed analytical memos for each. Emerging themes were confirmed in consultation with IO and categorized using an adapted version of the Social Determinants of Health (SDOH) framework. IO was part of the study team while MRT was external and unfamiliar with the study. Themes were organized into the following overarching domains: Cultural Factors [i.e., cultural attitudes toward weight loss], Environmental Factors [i.e., walkability, safety, or access to grocery stores and affordable healthcare], Individual Factors [individual and participant-specific characteristics such as lack of motivation, family health history, time constraints, and physical health barriers], Social Factors [i.e., social support and/or stigma], and Study Factors [Participants’ understanding of health interventions and resources]. Themes were further divided into subgroup categories of facilitators and barriers to provide a nuanced understanding of participants’ experiences.

Study method and results are reported following the Standards for Reporting Qualitative Research (SRQR) checklist [19].

## RESULTS

### Demographics

We invited 38 eligible participants from the parent EVO trial, and 26 of those participants completed qualitative interviews between April to August 2024. Included participants had completed the active phase of the intervention between April 2021 to August 2024. Just over half of the EVO trial participants (*n = 14*, 53.85%) were randomized into a condition receiving mHealth components with 12 brief coaching calls (APP), while the remaining were randomized to receive an individually delivered version of the Diabetes Prevention Program (DPP). Details about these conditions are published elsewhere [17].

Participants (*n = 26*) were adults aged 27 to 72 years (M = 50.3, SD = 10.1). Of these 26 participants, the majority (88.5%) were female, 76.9% identified as non-Hispanic or Latino, and 53.9% identified as Black or African American. Most of the participants (80.8%) completed a minimum of a 4-year college degree, 88.5% lived in Rural-Urban Area (RUCA) code level 1 (Metropolitan area core) [20], and 65.4% had an annual household income of $75,000 or higher. Participant characteristics are detailed in Table 1.

### Cultural Factors

#### Facilitators: Familial Support, Cultural Beliefs and Perceptions, and Traditional Dietary Habits

Family perceptions of weight and health emerged as a powerful motivator, as cultural beliefs about health and well-being were often reinforced through generational traditions. Many participants reported that family members, particularly parents or older relatives, emphasized the importance of maintaining a healthy weight and balanced diet. Family guidance provided practical and emotional support, reinforcing participants’ commitment to staying engaged with the program.

“And my parents, when I grew up, they really emphasized a lot on vegetables and stuff, so I was already eating a lot. We eat a lot of collard greens, a lot of kale, a lot of cauliflower, and stuff like that…” – PID 7306

Cultural expectations around body image also acted as a significant motivator for many participants. In the cultural contexts of some participants, thinness was associated with success, attractiveness, or self-discipline. For these participants, the desire to meet cultural ideals of body size often drove their pursuit of weight loss. This reinforced their dedication to engage in the intervention and pursue behavioral changes.

Traditional dietary practices played a dual role. While certain cultural dishes posed barriers due to their calorie density or preparation methods, others found ways to adapt traditional recipes to align with healthy eating practices. Participants who found ways to incorporate traditional foods into their weight management efforts reported greater success in sustaining healthy behaviors while maintaining cultural connections.

Barriers: Food as a Marker of Cultural Identity & Cultural Perceptions of Body Image and Weight Loss

Many participants described food as central to their cultural identity, making it difficult to modify dietary habits. One participant noted:

“*Growing up, food was a very social thing, especially in our culture. Everybody eats together, everybody has big meals together. We come together, and if you’re not eating, it’s offensive to whoever cooked or whoever invited you*.” - PID 7325.

Thus, participants frequently encountered pressures to eat certain foods during gatherings or celebrations, and rejecting food in these settings was often perceived as rude or ungrateful.. Similarly, modifying traditional dishes to align with healthier eating practices required effort and emotional negotiation, as participants feared being perceived as rejecting their culture. Some participants struggled to balance cultural expectations with their personal health goals, leading to difficulties in sustaining dietary changes.

Cultural perceptions of body image also contributed to engagement barriers. While thinness was valued in some cultural contexts, other participants described cultural norms that equated larger body sizes with health, prosperity, or attractiveness. This created uncertainty about weight loss goals and, in some cases, reduced motivation to engage in behavioral change.

### Environmental Factors

#### Facilitators: Access to Safe and Convenient Exercise Spaces and Healthy Food Environments

Participants described using their homes, neighborhoods, and community resources to stay active, noting that access to spaces made exercise easier to integrate into their daily routines. Some resources provided flexibility and convenience when gym access was limited.

“Yeah, I live in a really nice neighborhood, so my sport in the last 5 years has been pickleball, so we have nice courts, you know, within our neighborhood. So, I would play like 3 hours Saturday morning and 3 hours Sunday morning. With a good group of friends…it’s a good motivation to be active” – PID 7157

Participants also shared how the availability of affordable fresh produce, whole grains, and lean proteins enabled them to make healthier dietary choices. Some also described strategies they used to adapt their eating habits, such as controlling the types of food kept in their homes and practicing portion control.

“So, I definitely try to remove the snacks from the home. And…I changed the way I eat because…I loved sweets, but it’s like when you start eating fewer sweets, then sweets taste really sweet. So, starting to remove that stuff from the home and reducing the amount of access and sort of default behaviors was helpful.” - PID 7691

#### Barriers: Unhealthy Food Environments, Limited Access to Safe and Convenient Exercise Spaces

For participants living in rural areas or food deserts, the lack of access to stores offering affordable fresh produce or nutritious food further compounds these challenges. Remote work environments and busy schedules also disrupted meal preparation routines, leading to reliance on less nutritious convenience foods.

“It’s also hard to get good, nutrition-dense, healthy food because it’s a rural area. Fruits and vegetables, you buy them one day and you take them home, and the next day they’re bad because they have to travel so far, so it’s hard to get those. Then it’s a short growing season, so I tried to do a garden, but it’s the cold area, too, so it’s a short growing season. Then I wasn’t able to produce a lot of vegetables…” – PID 7325

In terms of physical activity spaces, some described feeling unsafe exercising outdoors due to environmental concerns, such as crime or poor infrastructure. Those in apartments described space restrictions that made it difficult to engage in home workouts. Additionally, long work hours and periods of high stress diminished participants’ motivation and energy to prioritize exercise, creating further obstacles to maintaining an active lifestyle. Financial constraints prevented some participants from affording gym membership or exercise equipment, further limiting their ability to stay active.

“I love the outdoors, but I’m in Florida, and once it gets a certain season…it’s so hot, and then…I’ve fallen several times running because sometimes you don’t notice and you trip on something…As I got older, I’m just like, I can’t deal with this. So…we did get a treadmill because we had one that somebody had given us, and then it broke down…” – PID 7321

Participants sought creative solutions to overcome environmental barriers, such as using additional online workout resources, preparing meals in advance, and finding alternative ways to stay active.

### Social Factors

#### Facilitators: Familial and Community Support, and Media Influence

Family support emerged as a significant source of emotional and practical assistance. Participants shared that family members not only encouraged them to pursue weight loss goals but also provided accountability and contributed to creating a supportive environment by assisting with tasks like meal preparation or childcare. Family-oriented activities, such as group walks or shared healthy meals, further strengthened their motivation to continue their efforts.

“Well, my sister knew about the study, I listed her name as a reference. She thought that was really great. I don’t think I told anybody at my workplace that I was on the study. My doctor knew about it because she had to sign off on it. She was absolutely she was in favor of it.” – PID 7064

Community-based resources, including gyms, fitness groups, and local wellness programs, played a key role in participants’ success. Structured programs, group workouts, and events such as triathlons fostered a sense of belonging and accountability. Additionally, support from coaches, therapists, and healthcare providers further enhanced participants’ ability to stay on track by offering guidance and encouragement.

Participants also noted that exposure to positive messages and success stories about health and fitness through social media, entertainment industries, and broader social trends reinforced their weight loss efforts. Several participants credited social media platforms with providing access to virtual fitness communities and resources, supporting their engagement with health initiatives.

#### Barriers: Lack of Familial and Community Support, Social Norms, and Stigmatization

Participants highlighted significant challenges stemming from living with family members or housemates who did not prioritize healthy lifestyles or eating habits. Examples included family members bringing unhealthy snacks into the home, a lack of emphasis on physical activity, and difficulties aligning family meal planning with individual dietary goals, especially for those with cooking and caring responsibilities.

“Just the whole when you have to cook for somebody who’s not on a diet and they don’t want to be on a diet, they don’t see why they have to lose weight just because you are. It really is kind of demotivating, let’s say I want to eat broccoli and meat just for that time, but I have to make fries and a steak and whatever. The temptation is really very high.” – PID 7306

Unsupportive family attitudes, including dismissive comments or a lack of understanding regarding participants’ weight loss efforts, also contributed to stress and undermined motivation. A lack of support manifested in other ways, such as peers dismissing the need for change, friends or partners inadvertently sabotaging efforts by offering participants unhealthy foods, and social gatherings centered around calorie-dense meals. The lack of a health-focused social circle made it difficult to stay committed to their goals and maintain accountability.

“Since the study, I’ve lost even more weight. People are like, oh, don’t get too skinny, your head is going to get too big…you’re going to disappear?” – PID 7181

Negative social perceptions of weight and weight loss were also significant barriers. Participants reported encountering stigmatizing beliefs, social pressures surrounding body image, and marketing messages promoting quick fixes, all of which contributed to self-doubt and diminished confidence in their ability to achieve long-term behavioral change. Cultural beauty standards and societal expectations regarding weight exacerbated feelings of inadequacy, particularly for those navigating social environments where appearance was highly scrutinized.

“Yes, so the societal norms. I just think that we’re just so heavily marketed to, especially in January, New Year’s resolutions and that thing. But now, even more so, the celebrities and how they look, they use that to sell all these quick, sometimes very expensive methods, almost unrealistic, and how they attain the way they look. They try to pass it on. It’s just so easy to get like that, and just because you look that way, doesn’t necessarily mean you’re healthy. But that’s how it comes across.” – PID 7166

### Personal Factors

#### Facilitators: Self-Motivation, Readiness, and Family Medical History

Many participants emphasized their determination that enabled them to persist in making healthier lifestyle choices, even in the face of challenges. A readiness to adopt new behaviors, including healthier diets and regular physical activity, was crucial to participants’ success. Many expressed feelings of empowerment and personal growth throughout the study, finding satisfaction in their ability to overcome barriers and maintain progress.

“… I felt like I could benefit from the extra support of having a health coach. So just my willingness to be committed to it. I just believed that I could be successful with some help…” – PID 7166

Participants also frequently cited their family’s medical history, particularly the prevalence of chronic conditions like diabetes, heart disease, and obesity, as a significant motivator for change. Concerns about these health issues and a desire to break generational cycles of poor health led many to prioritize weight loss and adopt healthier habits.

“…my mother has diabetes…I was overweight…I wasn’t considered prediabetic or anything like that. And I’ve been someone who’s been pretty active. My whole life, I played sports and different things like that. And so, it made me be like, oh my God, I need to do something because I don’t want to become diabetic. So, I started doing it…” – PID 7181

#### Barriers: Time Constraints, Physical Health Challenges, and a Lack of Self-motivation

Competing demands often made it difficult to prioritize exercise, meal planning, and other study-related activities, leading to inconsistent adherence to the study’s requirements and goals.

“So, the biggest issue that I had was because I was working so many hours. I was not getting in all of the workouts, the minutes that I needed to, which typically, I would have figured out how to squeeze that in. But just because of the demand, I was up under it. Basically, I woke up started working until I fell asleep. So, it was just time constraints…” – PID 7097

Physical health challenges also posed significant obstacles for a few. Chronic conditions, injuries, or physical disabilities limited some participants’ ability to engage fully in exercise. This often required these participants to adjust their approaches, which impacted their overall progress.

“… when I signed up for the study, I didn’t have any physical problems…I was only able to do that for about three weeks, and then my feet started hurting really bad. That’s why I felt like you all were wasting your time with me because I wasn’t able to do everything that was required of me” - PID 7201

Some participants also reported a lack of overall internal drive or willpower, which hindered their ability to stay on track with their weight loss goals.

### Study Factors

#### Facilitators: Accountability and Goal Setting & Supportive Coaches, and Resources

Setting concrete, achievable goals was a key factor in their success. Specific milestones made the process feel more structured and manageable, enabling participants to track progress and celebrate small achievements along the way.

“I’m a person that really needs help with accountability. It helped me determine or identify and make specific objectives, goals, and timelines; with meeting with that individual [health coach], at least I would know it would be once weekly, and give me more focus to get some of those goals initiated within that timeframe.” – PID 7016

The role of approachable and empathetic coaches was also highlighted as crucial. Participants appreciated the personalized guidance they received, which fostered a sense of connection and reassurance. Many noted that the coaches’ ability to address specific challenges and offer practical suggestions for improvement helped them maintain motivation. Participants also provided positive feedback regarding the study materials and tools, including informational resources, workbooks, apps and devices, food diaries, and activity logs. These materials not only enhanced their knowledge but also reinforced their commitment by helping them stay accountable.

#### Barriers: Tracking and Technology Challenges, Unsupportive Coaches, and Inadequate Resources

Several participants reported significant difficulties with food tracking and maintaining consistent portion control, describing the process as time-consuming and challenging. This negatively impacted participants’ ability to manage their eating habits effectively. Four participants also expressed frustration with the lack of culturally relevant food options within the tracking tools, which contributed to feelings of exclusion and dissatisfaction.

“I didn’t like the dietary app. I mean, I liked the lessons, but I did not like how I had to track my food. It was just tedious. It was not a user-friendly app.” – PID 7166

Technical issues with the study’s app and spreadsheet tracking feature of the DPP condition were also significant obstacles. Four participants reported problems with the app, including syncing issues and difficulty navigating its features. Nine participants found the spreadsheet system for logging progress to be complex and cumbersome.

Additionally, some participants expressed dissatisfaction with their coaching experience, citing a lack of personal connection, overly scripted interactions, and rushed calls. When coaching felt impersonal or insufficiently tailored to their needs, participants found it more challenging to stay motivated and engaged. Three participants expressed feeling undervalued during rushed or infrequent coaching calls, negatively impacting their focus on weight loss goals. This undermined participants’ confidence and engagement. For some, the absence of continuous monitoring and personalized feedback hindered their motivation and adherence to the program.

## DISCUSSION

This study explored factors that contributed to successful engagement, or otherwise, with a behavioral weight loss intervention from the participants’ perspectives. Although these factors are not only applicable to racial and ethnic minority groups, we set out to explore these understudied factors among these groups within an interventional trial because of the known disproportionate prevalence of overweight and obesity affecting them [21].

Unsurprisingly, themes that arose from this work included cultural, environmental, social, personal, and study-related factors. Consistent with extensive prior literature, participants in the present study felt more supported by favorable cultural traditions, unlimited environmental access to resources, positive social influence, personal motivation and readiness, and positive study-related experiences [22–26]. Participants also reported that these same factors constituted barriers to successful engagement, as previously reported in the literature.

All the participants had some level of college education, the majority lived in metropolitan areas as classified by RUCA [20], and two-thirds had a household income of $75,000 or higher. This finding may indicate that the participants in the parent study [17] are in the middle socio-economic class or higher. The parent study recruited participants via online advertisements into a remote, decentralized behavioral intervention using electronic devices. This may present a sampling bias towards recruiting participants who are well-educated and can interact with electronic media [27].

Participants mentioned the significant influence of cultural or family traditions on their dietary intake, which was a major part of the intervention. Food choices are not only shaped by individual factors such as taste, affordability, and social interactions, but also by people’s beliefs, family traditions, culture, and Faith [28]. In many cultures, food identity persists with individuals and is transferred through generations, even if they change their geographic location [29]. The participants of a previous study among Latina immigrants expressed a desire for weight loss programs that preserve traditional foods and include family [30]. Obesity treatments must acknowledge the intersection of cultural identity and health by adopting culturally sensitive approaches to help participants navigate cultural barriers, such as adapting traditional recipes and seeking familial support, to prioritize well-being.

The built environment directly impacts people’s access to healthy foods and their ability to engage in physical activity, ultimately affecting health [24]. Overall diet quality, which is a better estimation of diet-related risks [31], is affected by environmental access. Inadequate environmental access and natural disasters, when compounded with unfavorable social factors, pose significant barriers to successful behavioral enactment for weight loss treatments [32, 33]. While some studies consider environmental factors as predictors of variation in weight loss or moderators of the effectiveness of weight loss interventions [34, 35], most studies only focus on modifying individual factors and not environmental factors. Obesity treatment may benefit from interventions targeted at environmental modifications.

In addition to the positive influence of social factors on weight loss efforts, Ogden and Quirke-McFarlane [36] proposed a model for understanding the pathways of negative social support in weight loss efforts, including sabotage, feeding behavior, and collusion. Our findings support the double-edged influence of social factors and emphasize the need to incorporate this into behavioral interventions. Past interventions, including our study, usually have lessons that encourage participants to seek social support for their goals [7, 17]. To go a step further, interventions might integrate social support strategies shown to be effective and/or follow models of family-based or family-focused interventions often used in childhood obesity interventions [37–39].

Our findings show that social interaction is important, and this is consistent with findings from observational studies [40, 41], intervention studies that leverage social media [42], and other group-based interventions [43, 44]. However, a balance is often needed to make such support viable, whether it involves a known or unknown peer, a group that bears the burden of scheduling and privacy, or the question of whether online support can be as effective without the access burden of in-person interactions. Weight management trials have the additional barrier of managing and coordinating such nonorganic social support, but given its importance, particularly for some subgroups of the population, it is important we find ways to study the conditions in which social support is helpful.

Personal factors, including readiness, self-motivation, knowledge of family medical history, physical ability, and time constraints, were vital to participants’ engagement in the treatment, as established in previous studies [25, 45]. Notably, time constraint due to busy work schedules and family responsibilities is a recurring barrier to engagement in weight loss interventions in many studies [46, 47]. Previous studies suggested that individuals with initially high autonomous motivation may still find time to self-monitor and engage with the intervention, especially if they achieve some level of weight loss [48]. Further studies are needed to explore the correlation between autonomous motivation and perception of time commitment in weight loss interventions. Consistent with our findings, pain and physical discomfort are common reasons people with obesity do not engage in physical activity [49, 50]. Engagement barriers due to physical health may be addressed by providing a personalized, participant-centered approach to physical activity enactment [51].

Several components of the intervention, including access to educational tools, accountability, and personalized coaching experience, constituted both barriers and facilitators to participants’ engagement with the intervention. It is not uncommon for participants to value intervention components differently, which may be due to their specific needs and contexts [52]. A typical example is dietary self-monitoring; while some participants reported an overall positive experience, other participants with immigrant identities reported challenges, including not having access to culturally relevant food. While most weight management interventions incorporate personalized coaching experience, we suggest that participants may benefit more by extending this personalized experience to the design of intervention components.

Another notable intervention component that received mixed perception from participants is coaching. Personalized health coaching and participants’ perceived connection with the health coach affect participants’ experience and retention in behavioral weight loss interventions [26, 53]. However, the authors of another study argue that the impact of the participant-interventionist bond on intervention success may be secondary to other factors such as self-monitoring and early weight loss [53], further emphasizing the likely impact of participants’ specific needs and contexts on perception. Other factors that may affect coaching experience include scheduling difficulty and participants’ motivation, making it difficult to assess how coaching experience impacts success or how all the factors interact. Further studies are needed to assess how participants’ internal motivation and other personal factors moderate the relationship between coaching experience and intervention success.

The present study introduces a new perspective on the exploration of perceived barriers and facilitators to engaging in behavioral interventions delivered remotely, focusing on a nationally decentralized sample of participants with racial and ethnic minority identities. This is especially important due to the disproportionate obesity burden among these groups [1]. Furthermore, this study highlights the need to design intervention tools that are culturally relevant and inclusive to all participants, especially because participants’ specific needs and contexts seem to have a huge impact on perceived success. A major limitation of this study is the over-representation of female participants, which reflects the demography of the participants in the parent study and trials of weight management interventions in general [54]. Under-representation of male participants in obesity treatment trials could restrict the ability to understand their unique perspectives and limit generalization [55]. Furthermore, participants’ perceptions may be subject to recall bias, especially for those who participated in the interview about two years after the intervention.

## CONCLUSION

Perceived barriers and facilitators of engaging with remotely delivered behavioral weight loss intervention among racial and ethnic minority groups include cultural, environmental, social, personal, and study-related factors. Findings from this study emphasize the need to consider multiple factors in the design of behavioral weight-loss interventions. Participants had mixed perceptions about some factors, underscoring the likely impacts of personal needs on perceptions. Multi-level interventions, including some aspects targeting participants’ environments, and duly considering participants’ specific needs and contexts, may be a compelling strategy to improve the effectiveness of weight loss interventions.

## Figures and Tables

**Table 1:** Participant Characteristics

Characteristics		*n = 26*
Sex, n (%)	Female	23 (88.5)
	Male	3 (11.5)

Ethnicity, n (%)	Hispanic/Latino	6 (23.1)
	Not Hispanic/Latino	20 (76.9)

Race, n (%)	Black/African American	14 (53.9)
	White	4 (15.4)
	Mixed/Others	4 (15.4)
	Asian	3 (11.5)
	American Indian/Alaska Native	1 3.9)

Age (years)	Mean	50.3
	Standard Deviation	10.1

Education, n (%)	College graduate	21 (80.8)
	Some college/technical school	5 (19.2)

RUCA^1^ Code, n (%)	1 (Metropolitan area core)	23 (88.5)
	2 (Metropolitan area high commuting)	2 (7.7)
	7 (Small town core)	1 (3.9)

Household Income, n (%)	$75,000 or more	17 (65.4)
	$50,000 to less than $75,000	5 (19.2)
	$25,000 to less than $35,000	2 (7.7)
	$20,000 to less than $25,000	2 (7.7)

Study condition	APP	14 (53.9)
	DPP^2^	12 (46.2)

1: Rural-Urban Commuting Area Codes;

2: Diabetes Prevention Program

## Data Availability

De-identified data from this study are not available in a public archive. De-identified data from this study will be made available (as allowable according to institutional IRB standards) by emailing the corresponding author.

## Abbreviations

EVO: Elements Vital to Treat Obesity
DPP: Diabetes Prevention Program
SDOH: Social Determinants of Health
RUCA: Rural-Urban Commuting Area Codes
